# SIRT1 regulates Mxd1 during malignant melanoma progression

**DOI:** 10.18632/oncotarget.21457

**Published:** 2017-10-03

**Authors:** Fabiana M. Meliso, Danilo Micali, Camila T. Silva, Thaís S. Sabedot, Simon G. Coetzee, Adrian Koch, Fabian B. Fahlbusch, Houtan Noushmehr, Regine Schneider-Stock, Miriam G. Jasiulionis

**Affiliations:** ^1^ Ontogeny and Epigenetics Laboratory, Pharmacology Department, Universidade Federal de São Paulo - UNIFESP, São Paulo, SP, Brazil; ^2^ Department of Genetics, Ribeirão Preto Medical School, University of São Paulo, Ribeirão Preto, SP, Brazil; ^3^ Experimental Tumorpathology, Institute of Pathology, Friedrich-Alexander-Universität Erlangen-Nürnberg (FAU), Erlangen, Germany; ^4^ Department of Pediatrics and Adolescent Medicine, Friedrich-Alexander-Universität Erlangen-Nürnberg (FAU), Erlangen, Germany

**Keywords:** sirtuin-1 (SIRT1), MYC/MAX/MXD1 network, epigenetic regulation, melanoma progression, DNMT3B

## Abstract

In a murine melanoma model, malignant transformation promoted by a sustained stress condition was causally related to increased levels of reactive oxygen species resulting in DNA damage and massive epigenetic alterations. Since the chromatin modifier Sirtuin-1 (SIRT1) is a protein attracted to double-stranded DNA break (DSB) sites and can recruit other components of the epigenetic machinery, we aimed to define the role of SIRT1 in melanomagenesis through our melanoma model. The DNA damage marker, γH2AX was found increased in melanocytes after 24 hours of deadhesion, accompanied by increased SIRT1 expression and decreased levels of its target, H4K16ac. Moreover, SIRT1 started to be associated to DNMT3B during the stress condition, and this complex was maintained along malignant progression. *Mxd1* was identified by ChIP-seq among the DNA sequences differentially associated with SIRT1 during deadhesion and was shown to be a common target of both, SIRT1 and DNMT3B. In addition, *Mxd1* was found downregulated from pre-malignant melanocytes to metastatic melanoma cells. Treatment with DNMT inhibitor 5AzaCdR reversed the *Mxd1* expression. Sirt1 stable silencing increased *Mxd1* mRNA expression and led to down-regulation of MYC targets, such as Cdkn1a, Bcl2 and Psen2, whose upregulation is associated with human melanoma aggressiveness and poor prognosis. We demonstrated a novel role of the stress responsive protein SIRT1 in malignant transformation of melanocytes associated with deadhesion. *Mxd1* was identified as a new SIRT1 target gene. SIRT1 promoted *Mxd1* silencing, which led to increased activity of MYC oncogene contributing to melanoma progression.

## INTRODUCTION

Sirtuin-1 (SIRT1) is an NAD-dependent class III histone deacetylase belonging to the protein family of sirtuins. It has usually been related to gene silencing through its deacetylase action on histones and non-histone targets. SIRT1 is well known to be responsive to stress and participates in the establishment and development of several tumors, including prostate, lung, breast, ovarian, hepatocellular carcinomas, and melanoma [1, 2, 3, 4, 5, 6]. Nevertheless, the role of SIRT1 in malignant transformation is controversially discussed and seems to be dependent on cell type and microenvironment (as reviewed by [7, 8]). It can promote tumor suppression [9, 10], but can also trigger malignant progression [3, 5, 11 12].

Carcinogenesis is associated with specific mechanisms of intracellular stress status, such as oxidative stress, and its consequences, including epigenetic modifications and DNA damage [13, 14, 15, 16]. The induction of a DNA double strand break in an exogenous E-cadherin promoter CpG island resulted in the relocation of SIRT1 to the site of damage, along with other components of the epigenetic machinery, such as DNA methyltransferase 1 (DNMT1), DNA methyltransferase 3B (DNMT3B), and Enhancer of zeste homolog 2 (EZH2) [17]. In this regard, it was reported that SIRT1 can relocate from regions non-rich in CGs to areas with high levels of CGs to increase their methylation [18]. Oberdoerffer and coworkers showed SIRT1 relocation in embryonic mesenchymal cells treated with hydrogen peroxide [19]. Here, SIRT1 dissociates from repetitive sequences and oncogenes and associates to DNA break regions, resulting in transcriptional changes similar to those observed during aging. Thus, SIRT1 relocation in response to oxidative stress might have a crucial role in the establishment of aberrant epigenetic patterns associated with the development of pathologies, including cancer.

Particularly for melanomas, it was shown that increased oxidative stress leads to several mutations, which are related to tumor initiation and progression (reviewed by [20, 21, 22]). Previously, we reported that the induction of stress in non-tumorigenic melanocyte lineage melan-a via several cycles of cell adhesion impediment resulted in epigenetic reprogramming and malignant transformation [23, 24, 25, 26]. Through this protocol, different cell lines were established, such as pre-malignant 4C melanocyte lineage, non-metastatic 4C11- and metastatic 4C11+ melanoma cell lines. In this *in vitro* melanocyte malignant transformation model, we demonstrated that after anchorage blockade, there was an increased level of reactive oxygen species, especially superoxide anion, which contributes to increased expression of DNMT1, DNMT3B and global content of methylated cytosine [27, 25]. These results suggested an important role of epigenetic modifications caused by environmental changes for the initiation of malignant transformation [23, 24]. Our previous studies have also shown increased SIRT1 expression during the initial phases of melanoma progression [25].

One of the major signaling pathways involved in cancer progression is the MYC/MAX/MAD network, which is related to cell growth, proliferation, differentiation, and apoptosis (reviewed by [29, 30]). The components MYC (Avian myelocytomatosis viral oncogene homolog) and MAD (or MXD, MAX dimerization protein 1) have antagonistic action, leading to transcriptional activation or silencing of several genes, respectively, by competing and interacting with MAX protein binding domain [31]. *Myc* acts as an oncogene and is frequently overexpressed in many types of cancer [32, 33, 34, 35]. In addition, *Myc* has been described as a candidate target for melanoma treatment [36]. It was shown that SIRT1 binds to the C-terminal region of MYC, which leads to MYC-MAX association and its subsequent activation [37].

Considering the fact that sustained stress of melanocytes leads to their malignant transformation and taking into account the potential role of SIRT1 in melanoma progression, this work aimed to evaluate the role of SIRT1 in our melanoma transformation model. For the first time, we show an interaction between SIRT1 and MYC/MAX/MAD network in melanoma progression, highlighting the SIRT1 role in the regulation of important pathways related to cancer.

## RESULTS

### SIRT1 shows increased expression and associates with DNMT3B during cellular stress caused by melanocyte adhesion blockade

**Figure 1:**
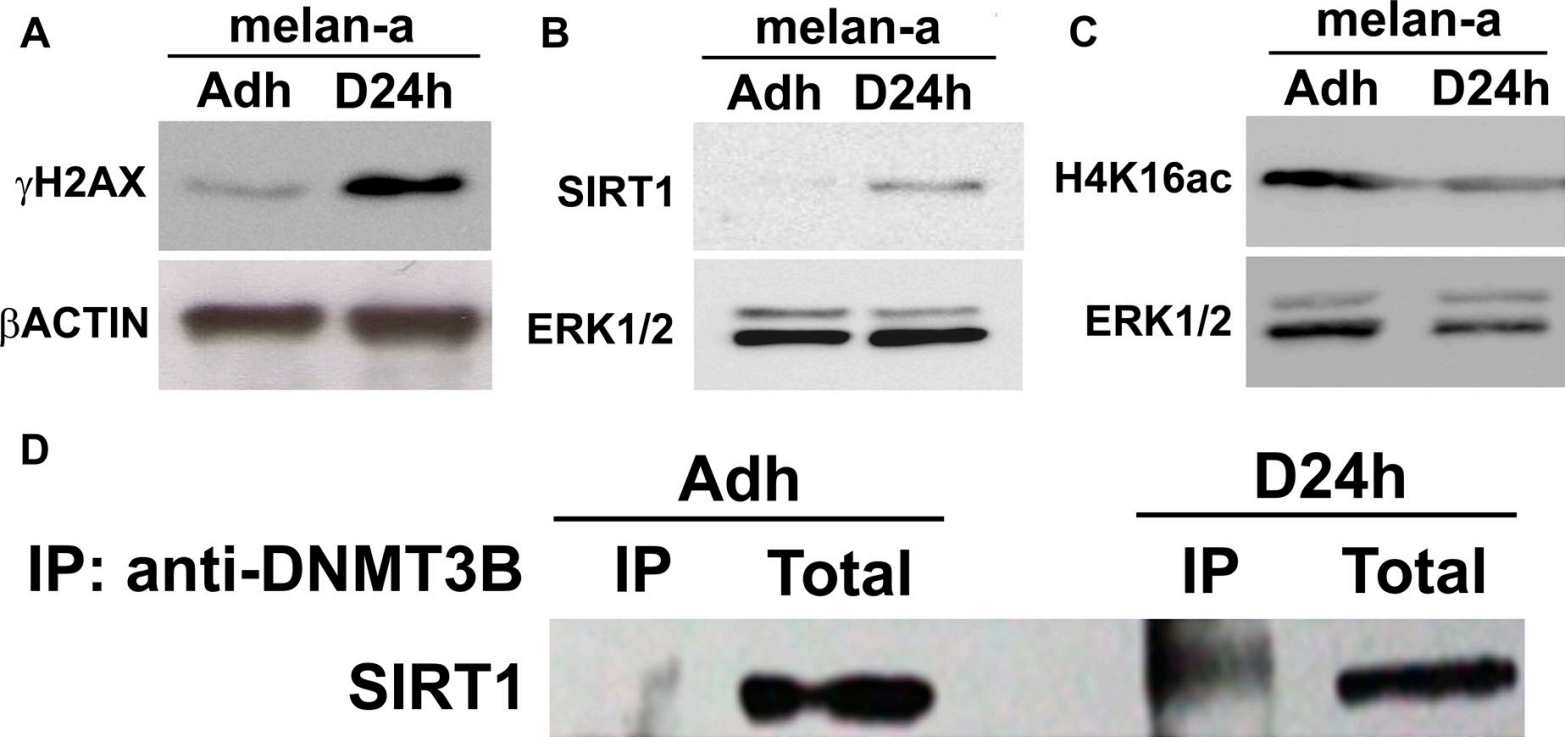
SIRT1 shows increased expression and associates with DNMT3B during stress condition The expression of γH2AX (**A**), SIRT1 (**B**) and H4K16ac (**C**) were evaluated by western blot in adhered (ma) and deadhered (D24h) melan-a melanocytes. The expression of β-ACTIN or ERK was used as endogenous control. The interaction between SIRT1 and DNMT3B (**D**) was determined by co-immunoprecipitation in adherent (ma) and deadhered (D24h) melanocytes. Total: Total protein extract used in IP assay; IP: immunoprecipitated protein fraction.

In a first step, we evaluated DNA damage and SIRT1 expression in melan-a melanocyte lineage subjected to adhesion blockade for 24 hours - a condition which, when repeated, results in malignant transformation [27]. An increase in γH2AX protein expression (Figure 1A), a characteristic mark of DNA double-strand breaks, as well as in SIRT1 protein expression (Figure 1B), was observed during deadhesion. This expression pattern indicates that deadhesion could lead to DNA damage and activates stress response via SIRT1. Indeed, as shown in Figure 1C, a decrease in the acetylation of lysine 16 of histone 4 (H4K16ac), a target of SIRT1, was observed during anchorage blockade, suggesting the increased SIRT1 expression could be associated with an increase in its deacetylase activity. Moreover, SIRT1 becomes associated with DNMT3B during deadhesion (Figure 1D).

### The cellular stress caused by melanocyte adhesion blockade leads to relocation of SIRT1 to DNA sequences associated with cell survival, proliferation, and migration

**Figure 2:**
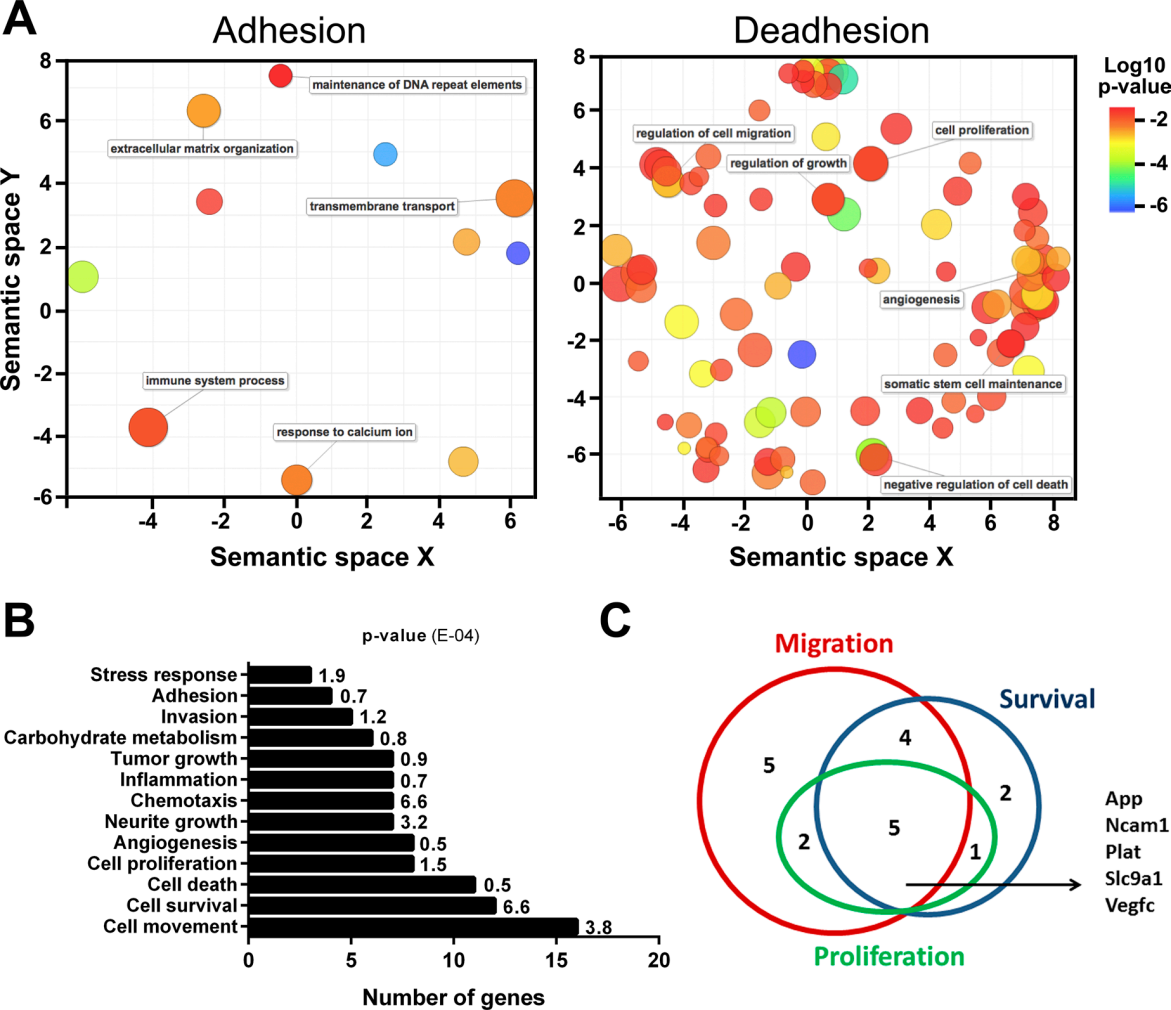
Under cellular stress, SIRT1 relocates to DNA sequences containing genes related to migration, survival, and proliferation (**A**) Semantic similarities of Gene Ontology (GO) terms for the genes related to normal adhesion condition of melanocytes. The scatterplot shows the representative GO clusters in two-dimensional spaces. Bubble color indicates the *p*-value and the size indicates the frequency of GO terms. More semantically similar GO terms are closer in the plot. The mainly pathways found in adhesion were extracellular matrix organization, transmembrane transport and immune system process. The main pathways found under stress condition (deadhesion) were regulation of cell migration, proliferation, angiogenesis, negative regulation of cell death, regulation of growth, among others. (**B**) Biological pathways related to genes in which SIRT1 is associated during deadhesion. (**C**) Venn diagram showing the number of genes related to migration, survival and proliferation in which SIRT1 has associated to during deadhesion.

**Figure 3:**
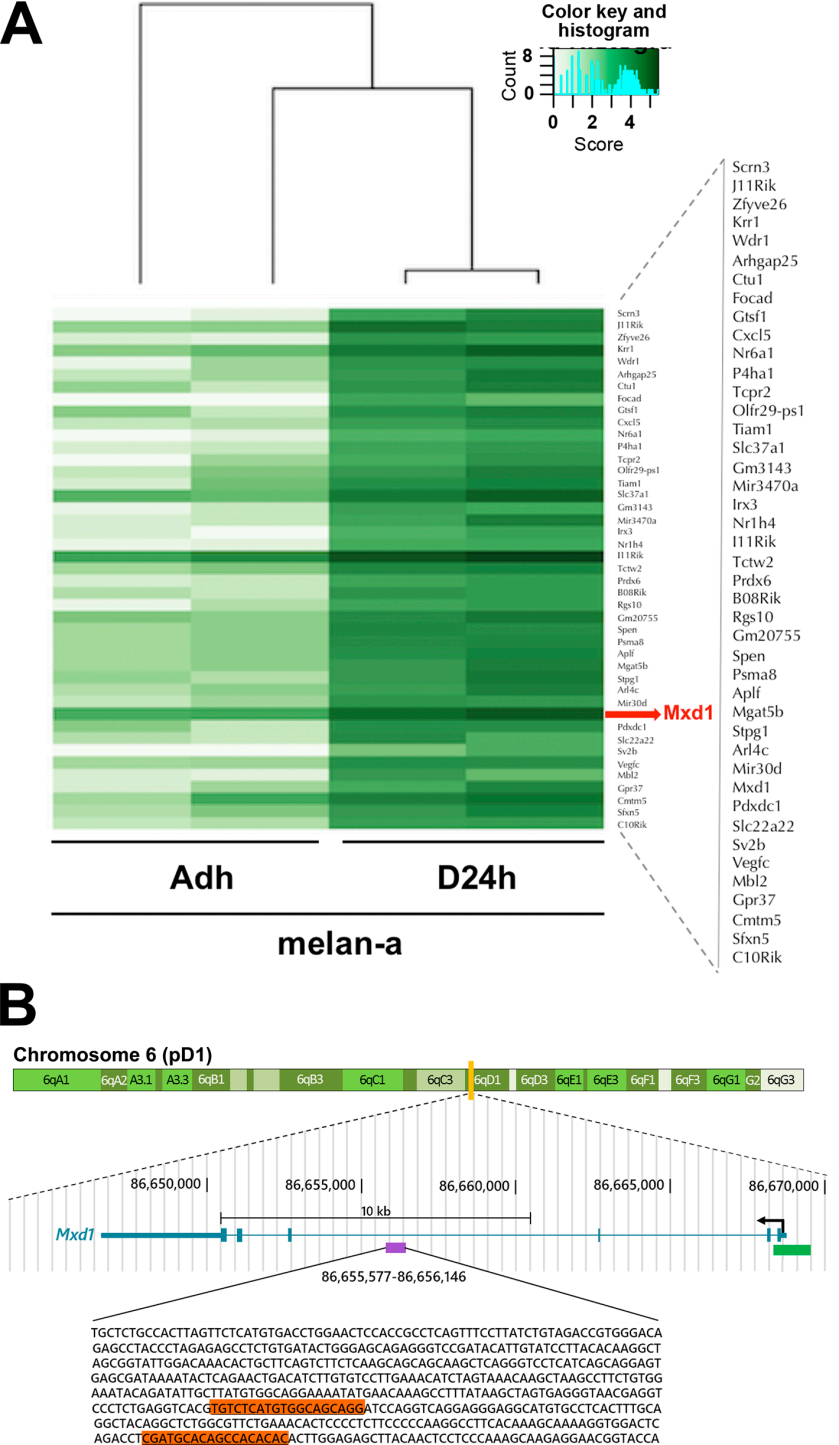
Increased SIRT1 association to *Mxd1* during stress condition (**A**) Heatmap showing the intensity in which SIRT1 was found to bind to Mxd1 DNA sequence in adhered (AD) and deadhered (D24h) melan-a melanocytes. (**B**) Mouse gene Mxd1 sequence. The purple rectangle represents the intronic region of the Mxd1 sequence where SIRT1 was differentially associated to before and during stress condition. The oligonucleotide sequence of the peak is represented below, where the orange rectangle represents the regions over which the primers forward and reverse were designed to confirm SIRT1 and DNMT3B association to Mxd1 sequence, by Real time-qPCR. The green rectangle represents a CpG island where Mxd1 transcription start site lies on. The blue schedule represents complete Mxd1 gene, located in the chromosome 6. Figure modified from UCSC Genome browser (https://genome.ucsc.edu/).

The SIRT1 relocation was analyzed by ChIP-seq to determine the DNA sequences where SIRT1 is bound during deadhesion compared to adhered melanocytes. The results showed that SIRT1 binds to a greater number of DNA sequences during the stressful condition compared to normal adhesion conditions (Supplementary Table 1). Our *in silico* analysis showed that in normal condition, SIRT1 tends to increase its association with DNA sequences next to genes related to pathways such as immune system process, transmembrane response and extracellular matrix organization (Figure 2A and Supplementary Table 2). While in the stress situation, SIRT1 has presented higher concentration value for sequences next to genes related to pathways involved in the establishment of cancer phenotype, like those related to migration, proliferation, angiogenesis, stress response, adhesion, invasion, carbohydrate metabolism, tumor growth, inflammation, chemotaxis, cell death, and cell survival (Figure 2A–2B, Supplementary Table 3). Among those pathways, our *in silico* analysis identified five SIRT1-associated genes (*App*, *Ncam1*, *Plat*, *Slc9a1* and *Vegfc*) that were concomitantly involved in the cellular processes migration, survival and proliferation (Figure 2C), further emphasizing the role of SIRT1 in the acquisition of malignant characteristics during cellular stress. Interestingly, an intronic region of the *Mxd1* gene (represented by the purple bar in the Figure 3B), an important modulator of MYC function [38], is among the sequences to which SIRT1 was found to be associated during stress condition (Figure 3A).

### SIRT1 regulates Mxd1 during melanoma progression

**Figure 4:**
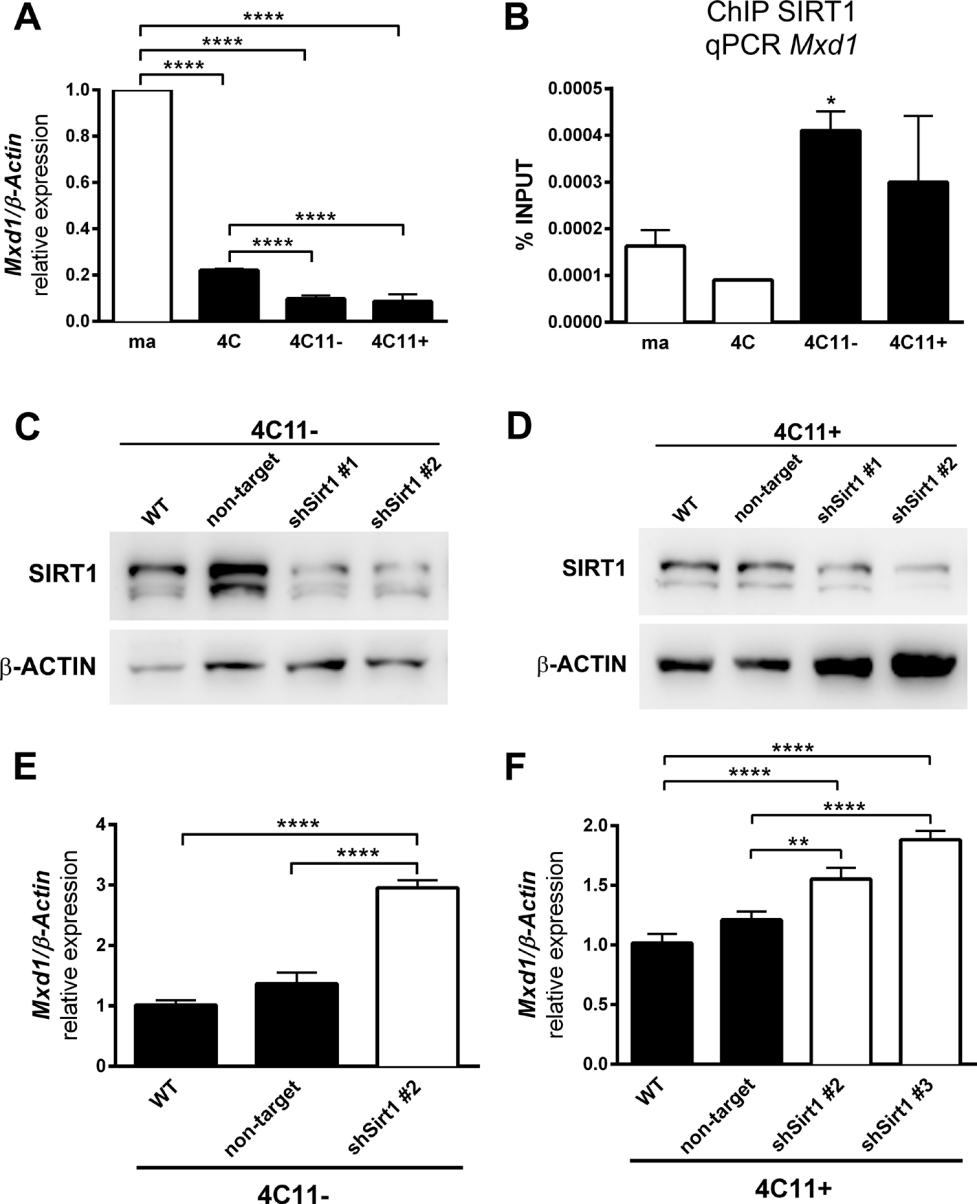
SIRT1 regulates *Mxd1* during melanoma progression (**A**) Mxd1 expression in cell lines representing different phases of melanoma progression determined by RT-qPCR. (**B**) Association of SIRT1 to Mxd1 gene along malignant transformation was evaluated by ChIP. SIRT1 protein expression in non-metastatic 4C11- (**C**) and metastatic 4C11+ (**D**) cell lines after stable SIRT1 silencing using shRNA analyzed by western blot. Mxd1 expression in 4C11- (**E**) and 4C11+ (**F**) melanoma cell lines silenced for SIRT1 evaluated by RT-qPCR. ma: melan-a melanocyte lineage; 4C: pre-malignant melanocyte lineage; 4C11-: non-metastatic melanoma cell line; 4C11+: metastatic melanoma cell line; WT: wild type; Non-target: non-target shRNA control; sh#: SIRT1 silenced clones. ^*^p < 0.01, ^**^p < 0.001, ^****^p < 0.00001.

Since SIRT1 participates on stress response [18, 19], has elevated expression, increased *Mxd1* association in melanocytes submitted to stress condition, and is usually involved in gene silencing, as expected for histone deacetylase, we suggest that SIRT1 could participate in *Mxd1* repression along melanocyte malignant transformation. To test this hypothesis, we evaluated *Mxd1* gene expression by RT-qPCR. As shown in Figure 4A, the *Mxd1* expression decreased along melanoma progression, being significantly reduced in pre-malignant 4C melanocytes and both non-metastatic 4C11- and metastatic 4C11+ tumor cell lines compared with parental melan-a melanocytes. By ChIP assay, using primers targeting the same *Mxd1* sequence found to be enriched in ChIP-seq (Figure 3B, primer sequences highlighted in orange), we showed that SIRT1 was more associated with *Mxd1* in melanoma cell lines (non-metastatic 4C11- and metastatic 4C11+ cells) compared to non-tumorigenic ones (melan-a melanocytes and pre-malignant 4C melanocytes) (Figure 4B). To further evaluate the effect of SIRT1 on *Mxd1* expression, the melanoma cells 4C11- and 4C11+ were stable silenced for SIRT1 (Figure 4C and 4D). Sirt1 silencing increased expression of *Mxd1* in both cell lines compared to non-silenced cells (Figure 4E and 4F), pointing to a *Mxd1* repression by SIRT1 in melanoma cells.

### Demethylating agent treatment reverses Mxd1 repression

**Figure 5:**
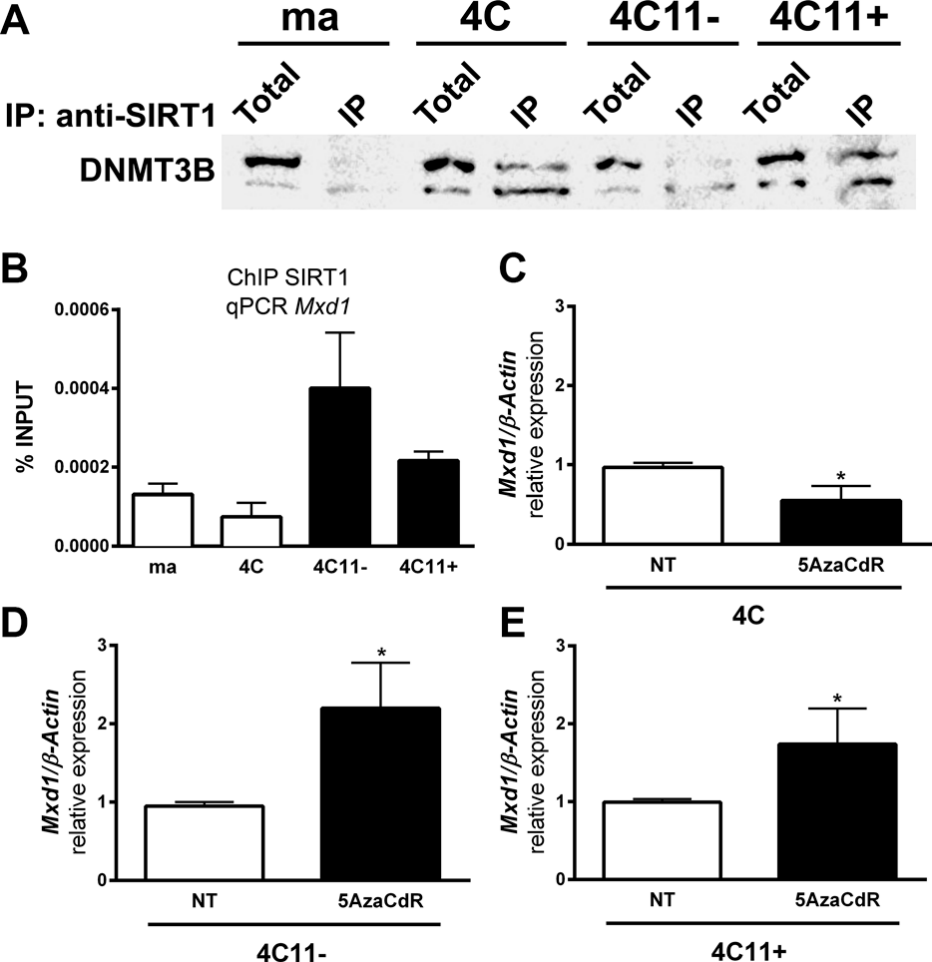
Demethylating agent treatment reverses *Mxd1* repression by SIRT1 (**A**) The interaction between SIRT1 and DNMT3B determined by protein co-immunoprecipitation assay. (**B**) Association of DNMT3B to Mxd1 gene along the malignant transformation was evaluated by ChIP. Mxd1 expression after treatment for 48h with 10 μM 5AzaCdR analyzed by RT-qPCR in 4C (**C**), 4C11- (**D**) and 4C11+ (**E**) cell lines. ma: non-tumorigenic melan-a melanocytes, 4C: pre-malignant melanocytes, 4C11-: non-metastatic melanoma cell line, 4C11+: metastatic melanoma cell line. Total: total protein extract used in IP assay; IP: immunoprecipitated protein fraction. ^*^p < 0.01.

As shown above, SIRT1 formed a new complex with DNMT3B during melanocyte deadhesion. To evaluate if this SIRT1-DNMT3B complex is maintained along melanoma progression, we performed protein co-immunoprecipitation. In fact, protein-protein interaction between SIRT1 and DNMT3B was observed both in 4C pre-malignant melanocytes and in 4C11- and 4C11+ tumor cells (Figure 5A). Interestingly, DNMT3B was found to be associated with *Mxd1* gene at the same site where SIRT1 interacts, since we have used the same oligonucleotides designed for the sequence of *Mxd1* peak where SIRT1 has associated during stress condition (Figure 3B, primer sequences highlighted in orange). Similar to SIRT1, DNMT3B was stronger associated with *Mxd1* in the tumor cell lines (Figure 5B). Moreover, *Mxd1* repression was reversed after treating tumor cell lines with the demethylating agent 5-Aza-2′deoxycytidine (Figure 5C–5E). Analyses by pyrosequencing of *Mxd1* promoter showed no significant difference in DNA methylation status, at least in this evaluated DNA sequence (Data not shown).

### Down regulation of Mxd1 favors MYC activation

**Figure 6:**
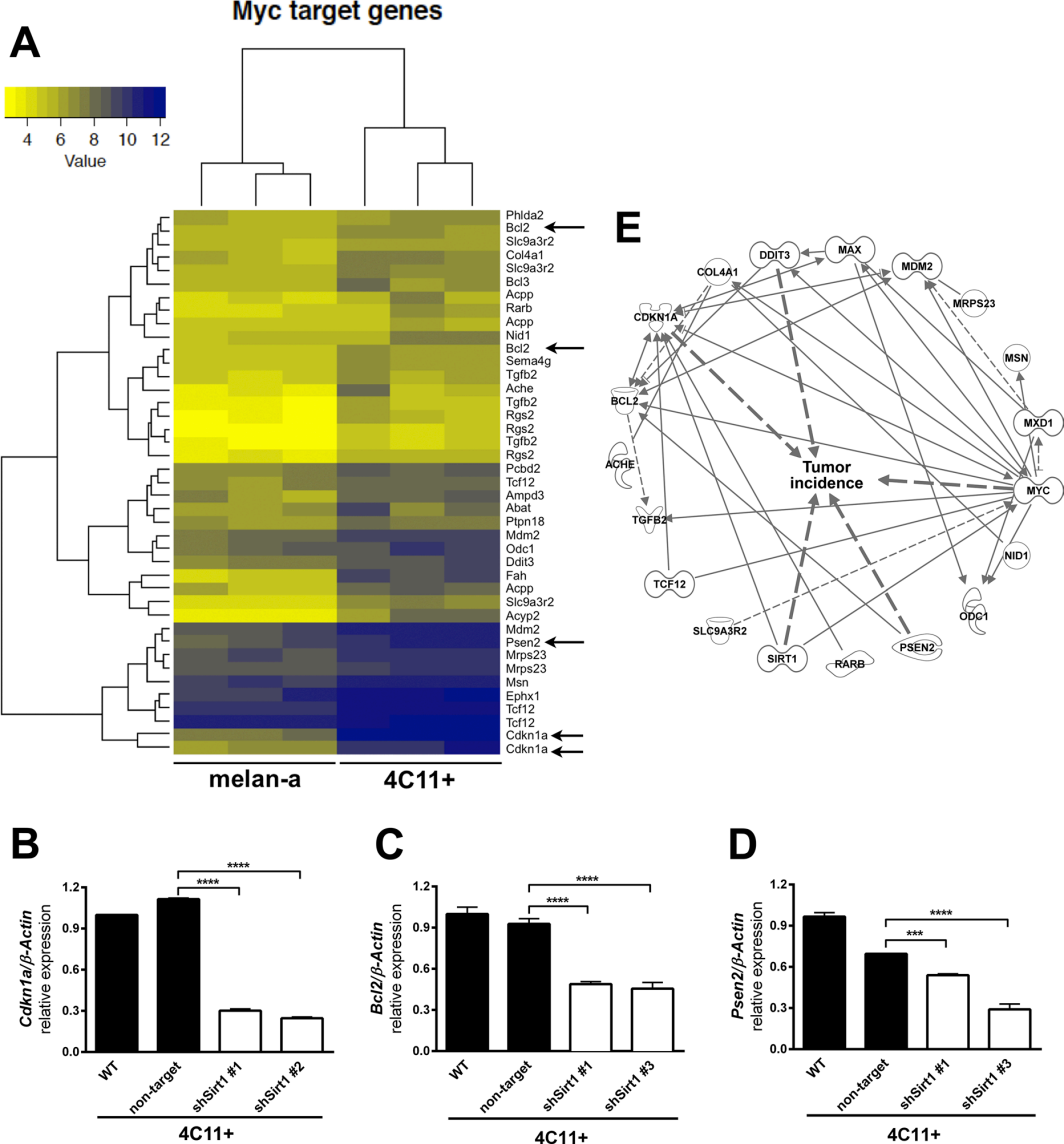
Down-regulation of SIRT1 leads to an increase of *Mxd1* expression and subsequent downregulation of MYC target genes (**A**) Heatmap showing increased expression of MYC targets in 4C11+ melanoma cells compared to melan-a melanocytes obtained by microarray assays (color key chart with respective values is given). Arrows indicate Cdkn1a, Bcl2 and Psen2 as some of MYC targets in 4C11+ melanoma cells. Expression of Cdkn1a (**B**), Bcl2 (**C**) and Psen2 (**D**) was determined by RT-qPCR in non-metastatic 4C11- and metastatic 4C11+ melanoma cell lines, control and silenced for SIRT1. (**E**) Illustration of the interrelation among MYC and SIRT1 target genes altered in our melanoma model by Ingenuity functional pathway analysis (IPA^®^) graphical database of networks of interacting genes (Ingenuity Knowledge Base, IKB^®^). Only genes with interactions are displayed and were further subjected to the built-in “Grow to Diseases & Functions” feature to detect genes related with “tumor incidence”. WT: wild type; non-target: non-target shRNA control; sh#: SIRT1 silenced clones. ^***^p < 0.0001, ^****^p < 0.00001.

It is known that MXD1 competes with MYC oncoprotein for binding to MAX (MYC associated factor X). Since *Mxd1* is downregulated by SIRT1 in 4C11- and 4C11+ tumor cell lines (Figure 4E and 4F), we have postulated that MAX could become more available to MYC binding in these melanoma cells. If a larger amount of MYC binds to MAX, we would expect an increased activation of MYC target genes. Among MYC targets, there are genes involved in tumorigenic processes, such as *Cdkn1 (Cyclin Dependent Kinase Inhibitor 1A)*, *Bcl2 (B-cell lymphoma 2)* and *Psen2 (Presenilin-2)*. In our melanoma model, the expression of these genes is increased in the tumor cell lines (Figure 6A) and, as hypothesized, there was a significant reduction of *Cdkn1a*, *Bcl2* and *Psen2* expression in SIRT1-silenced 4C11+ melanoma cell line (Figure 6B–6D, respectively) compared to non-silenced cell lines. Together, these results show that SIRT1 is triggering MYC target gene expression by inhibiting MXD1 function, which may contribute to cancer phenotype acquisition. In line with this finding, *in silico* analysis of gene interactions between *Sirt1, Myc* and *Max* revealed several genes linked to tumor incidence (Figure 6E).

### Decreased levels of *Mxd1* and increased levels of Myc-target genes in human melanomas are related to poor prognosis

**Figure 7:**
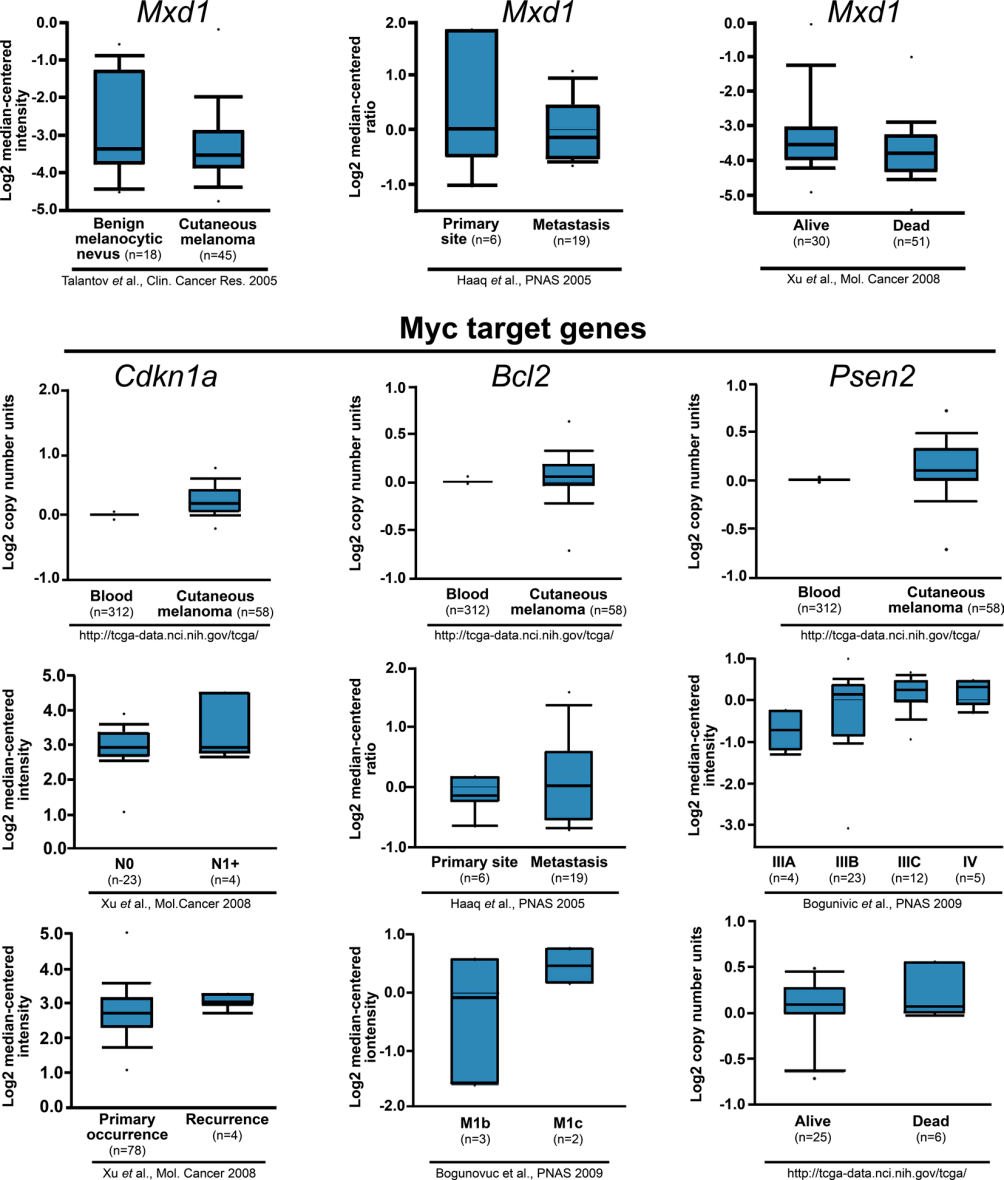
Decreased levels of *Mxd1* and increased levels of Myc-target genes in human melanomas are related to poor prognosis Data obtained from Oncomine database [40, 41, 42, 43] are shown across multiple independently published microarray studies as indicated. Boxes represent the interquartile range (25th–75th percentile). The bars denote the median. *P* <-1E4

From the Oncomine microarray public database analysis for human tumors [39], corroborating the results described above, *Mxd1* seems to be reduced in melanoma cells and in metastatic cells, while Myc target genes are increased in cutaneous melanoma and seem to be associated with a poor prognosis (Figure 7).

## DISCUSSION

Besides many advantages of existing mouse melanoma models, there are specific shortcomings regarding their recapitulation of natural tumor progression, from increasing proliferation to invasion and metastasis [44]. Our *in vitro* melanocyte malignant transformation model based on repeated cycles of anchorage blockade allows studying different stages of melanoma progression from normal melanocytes to non-metastatic and metastatic melanoma cells [27]. We do not use any carcinogenic agent or genetic engineering as stimulus, as the natural ability to survive anchorage blockade is a characteristic for disseminating tumor cells and finally leads to tumor transformation [45].

Previously, we have identified massive epigenetic alterations along tumor progression in our model [25]. Particularly, DNA methyltransferases were deregulated after anchorage blockade as a result of increased levels of superoxide anion [27, 28]. Particularly, we observed a SIRT1 up-regulation in our melanoma progression model [25]. Similarly, Ohanna and coworkers reported SIRT1 overexpression in resistant BRAF^V600E^-mutated melanoma cells [6]. The role of the deacetylase SIRT1 in cancer is not clearly understood and seems to be dependent on cell type and stimulus. To unravel the role of SIRT1 over-expression after anchorage blockade, we aimed to identify novel SIRT1 targets in different stages of melanoma development. For the first time, we unraveled that SIRT1 recruits DNMT3B and relocates to *Mxd1* gene under stress. MXD1 is a MYC inhibitor, competing with MYC for the binding of MAX co-activator. MYC/MAX/MXD1 network has been shown to play a major role in the development of neuroblastoma and melanoma [36, 46]. A high c-MYC and SIRT1 protein co-expression has been demonstrated to be associated with malignant transformation of specific colorectal cancer subtypes [47]. Inversely, Li and coworkers reported a positive regulation of SIRT1 by the c-MYC pathway in acute myeloid leukemia [48]. Since in our model SIRT1 represses *Mxd1* gene expression, we suggest that MYC is activated in melanoma progression. As MYC is closely associated with tumor aggressiveness such as self-renewal capacity [49], proliferation [6] and epithelial to mesenchymal transition [50], we hypothesize that this newly identified interaction might lead to early initiation and finally to tumor progression in our model. Furthermore, our data suggest a new mechanism explaining how SIRT1 inactivates gene expression through recruitment of DNMT, i.e. by recruitment of DNMT3B to the *Mxd1* gene promoter. DNMT3B is a *de novo* DNA methyltransferase, that is responsible for establishing new methylation patterns and is involved in gene silencing [51, 52]. Our data indicate that SIRT1 and DNMT3B co-participate in *Mxd1* epigenetic silencing in melanoma.

Our ChIP-seq data showed a SIRT1/*Mxd1* interaction already after 24 hours of anchorage blockade. Surprisingly, the ChIP assay along melanoma progression stages did not confirm this interaction in pre-malignant 4C melanocytes, but SIRT1/*Mxd1* interaction was remarkably evident at later stages. This genomic interaction might reflect high methylation dynamics along melanoma transformation and progression. The concept of high plasticity in methylation/demethylation in cancer has been highlighted very recently by Bell and coworkers [53] and Vizoso & Esteller [54]. Cells that are exposed to stressful conditions, like ROS induction by anchorage blockade in our model, need to adapt to these environmental alterations, which is mainly achieved by epigenetic reprogramming [55]. Indeed, SIRT1/DNMT3B complex was verified after 24h of anchorage blockade and at late stages of melanoma progression, but not at the pre-malignant 4C cells, a result that we aforementioned for SIRT1/*Mxd1* interaction. Corresponding to this, treatment of cells with DNMT inhibitor led to a continuous increase in *Mxd1* expression along melanoma progression, suggesting sequential acquisition of aberrant epigenetic marks. Moreover, Menssen and collaborators [56] found a positive feedback loop between SIRT1 and MYC activation in colorectal cancer. They described that SIRT1 increases transcription activity of c-MYC, whereas c-MYC induces SIRT1 deacetylase activity. As a limitation of our study, we have to take into account that ChIP-seq showed 500 bp long DNA binding region for SIRT1 at *Mxd1* gene, whereas the ChIP assay verified only a smaller 100 bp DNA sequence in this region and we cannot exclude sub-sequential stronger or transient binding of SIRT1 along the different stages of tumor progression. Depending on the ROS-induced DNA damage, the SIRT1 relocation at *Mxd1* sequence and stress-induced SIRT1 recruited repressive protein complexes might vary along the different steps of melanoma progression. Altogether, we give experimental evidences that in our melanocyte malignant transformation model, based on sustained anchorage blockade SIRT1, is a major player for the acquisition of an aggressive phenotype. We propose a new mechanism of MYC oncogene activation by SIRT1-dependent epigenetic silencing of *Mxd1* gene.

Considering the reversibility of epigenetic changes, the participation of SIRT1 and other epigenetic components in reducing *Mxd1* expression and in increasing MYC oncogenic activity might have prognostic and therapeutic potential in melanoma.

## MATERIALS AND METHODS

### *In vitro* melanoma progression model

The studies were performed using an *in vitro* malignant melanoma progression model, previously established by our research group [27]. In this model, the melanocyte malignant transformation occurs without using physical or chemical carcinogenic agents and without genetic manipulation. The non-tumorigenic melanocyte lineage melan-a, derived spontaneously from epidermal melanoblasts of mouse embryos [57], was submitted to a sustained stress situation, represented by cycles of successive adhesion blockade. The melan-a cells, that in normal condition grow adherent to the plate ground, were maintained in suspension for 96 hours. As expected for immortalized non-tumorigenic cell line, the vast majority of cells died by *anoikis* after 96 hours in suspension. After this time, the few surviving cells were plated again in adherent condition. Those cells were called 1C (subjected to one cycle of anchorage blockade). When the plate achieved 80% of cell confluence, the cells were trypsinized and replated in deadhesion condition for 96 hours and the surviving cells were plated again in adherent condition and called 2C. These steps were repeated a third and fourth time, giving rise to 3C and 4C cell lines, submitted to three and four cycles of deadhesion, respectively. Subsequently, 4C cells were subjected to a fifth cycle of anchorage blockade for 96 hours and the surviving spheroids were submitted to a limiting dilution. After this, all randomly selected clones (among them 4C11-) were shown to be tumorigenic when injected into syngeneic mice and some of them were able to form metastatic colonies in the lung when injected in the caudal vein (as 4C11+). Besides forming tumoral colonies in the lung after intravenous inoculation, metastatic foci in axillary lymph nodes, confirmed by histological analysis, were found after subcutaneous inoculation of 4C11+ cells, demonstrating its metastatic phenotype. All the lineages derived from melan-a have shown stable phenotype, since they have maintained their morphology, expression of a panel of genes and proteins, and the same behavior in functional assays (such as proliferation, migration, invasion, anoikis resisitance, etc) along time.

### Cell culture

The cell lines used in this study were the non-tumorigenic melan-a melanocytes, the pre-malignant 4C melanocytes, the non-metastatic 4C11- melanoma cell line, and the metastatic 4C11+ melanoma cell line. The cells were cultured in RPMI pH 6.9 (Gibco, Carlsbad, CA), with 5% fetal bovine serum (FBS) (Gibco) and, just for melan-a, 200 nM phorbol 12-myristate 13-acetate – PMA (Amresco) was added. The PMA was added to the medium only for melan-a because it is necessary for proliferation and survival of cultured non-tumoral melanoblasts and melanocytes. Melan-a cells do not form tumors in syngeneic or athymic mice even after a long time being cultured in the presence of PMA (Bennett, Cooper, and Hart 1987). On the other hand, as described for melanoma cells, the cell lines obtained after four 96-hours cycles of adhesion blockade were capable of proliferating and avoiding senescence in the absence of PMA. All cell lines were grown at 37°C, under humidified atmosphere with 5% of CO_2_ until utilized in the experiments. Unless mentioned, all cell lines were cultivated in adherent conditions.

### Western blot

Protein extracts, prepared with RIPA buffer (50 mM Tris HCl (pH 7,4); 50 mM NaCl; 1% NP40 10%; 0.5% sodium deoxycolate 10%; 0.1% SDS 1%) containing protease inhibitors (PMSF 1 mM, Na_3_VO_4_ 1 mM, pepstatin 10 mg/ml, leupeptin 10 mg/ml, aprotinin 10 mg/ml), were separated by electrophoresis in SDS-PAGE polyacrylamide gel and transferred to a PVDF membrane. Then, each membrane was blocked for 40 minutes in a solution with 5% of non-fat milk. Next, primary antibody was added to the membranes and incubated according to manufacturer’s instructions. The membranes were washed and the secondary antibody was incubated for one hour, according to manufacturer’s instructions. Primary antibodies used were: anti-phospho-H2AX (Ser139) (Merck Millipore, 07–164), anti-SIRT1 (Abcam, #Ab12193), anti-DNMT1 (Imgenex, IMG-261A), anti-DNMT3B (Active Motif, #39207), anti-H4K16ac (Millipore, Temecula, CA, #07–329). After secondary antibodies incubation (KPL), the membranes were washed with TBS-T, detected with chemiluminescent SuperSignal (ThermoScientific) and visualized by luminescence reading device UVITEC (Cambridge, www.uvitec.co.uk).

### Protein co-immunoprecipitation

The association between SIRT1 and DNMT3B proteins was verified by protein co-immunoprecipitation assay. For this purpose, Protein G – agarose beads (10007D, Life Technologies) were used and the assay was performed according to the manufacturer’s instructions. Immunoprecipitation was performed with anti-SIRT1 (Abcam, #ab12–193), anti-DNMT1 (Imgenex, IMG-261A) or anti-DNMT3B (Active Motif, #39207), following datasheet specifications. The samples were used for subsequent analyses following western blot procedure.

### Chromatin immunoprecipitation (ChIP)

ChIP assay was performed with Active Motif kit (ChIP-IT^®^ High Sensitivity, catalog no. 53040), according to the manufacturer’s instructions. The ChIP assay was performed in independent duplicates.

### Chromatin immunoprecipitation followed by DNA sequencing (ChIP-seq)

ChIP-seq assay was performed with Active Motif kit (ChIP-IT^®^ High Sensitivity, catalog no. 53040), according to the manufacturer’s instructions. ChIP-seq assay was performed in independent duplicates. DNA from ChIP was prepared from melan-a melanocytes and from melan-a melanocytes submitted to anchorage blockade during 24 hours, as described by ChIP-IT^®^ High Sensitivity (Active Motif – 53040) protocol. After ChIP, enrichment for known targets was verified with INPUT (total DNA extract) and IgG samples by qPCR assay before sequencing. For pPCR reactions, we have used 1/10 input dilution. The qPCR results were normalized with the percentage of input amplification (sample signal value from IP assay was divided by input sample signal value). The DNA samples immunoprecipitated with anti-SIRT1 have been sequenced on a SOLiD4 machine from Life Technologies (Life Technologies, Carlsbad, CA, USA). Libraries for ChIP-seq were prepared following protocols recommended by SOLiD4. For alignment and mapping, the LifeScope mapper was used to map the reads across reference GRCm38/mm10 mouse genome. Then, Samtool package was used to sort and to remove the duplicates reads to improve specificity [58]. The reads quality was verified by FASTQC and the good mapping quality scores were considered Phred values ≥ 20 [59]. From BAM files alignment, Peak Caller MACs (Model basic analysis of ChIP-seq) was used to find regions in the genome with a significant number of mapped reads [60]. After normalizing and eliminating background, the redundant peaks were removed. Next, we used the open source software package Diffbind (Differential binding analysis of ChIP-seq peak data) from Bioconductor to count, adjust the contrast and to analyze the DNA sequences where SIRT1 was differentially bound [61]. DSEQ2, a Bioconductor package [62], was used to identify ChIP peaks associated with SIRT1. Finally, the packages Genomic Ranges and org.Mm were used to identify the gene closest to each of those peaks, considering GRCm38/mm10 mouse as the reference genome [63].

### Gene Ontology (GO) analysis

The GO analysis was performed to evaluate the mainly pathways that the closest genes to the peaks where SIRT1 has associated in normal and suspension condition were involved. For this, firstly we have used DAVID tool to obtain GO terms and *p*-values for each gene, according the procedure recommended [64, 65]. Then, we have used the obtained GO terms and *p*-values (less or equal to 0.05) for REVIGO analysis. Based on algorithms that reduce redundance, REVIGO finds a subset of representative and non-redundant GO terms as described previously [66]. The results were represented by two scatterplots, one for adhesion and other for deadhesion condition, showing the representatives clusterings remaining after the redundance reduction. More semantically similar GO terms are closer in the plot. The bubble size indicates the frequency of the GO term, while the color represents the *p*-value according to legend.

### *In silico* functional pathway analysis

For *in silico* functional pathway analysis of pathways related to SIRT1-associated genes, as well as interaction analysis of SIRT1/MYC/MAX we used Ingenuity Pathway Analysis (IPA) software (Qiagen, Redwood City, CA, USA), as previously described [67].

### 5-AzaCdR and TSA treatment

The compound 5-AZA-2′deoxycytidine (5-AzaCdR) is an inhibitor of DNA methylation, while Trichostatin A (TSA) inhibits HDACs of class I, II and IV. To evaluate the effect of methylation and histone modification on gene expression, the melan-a, 4C, 4C11- and 4C11+ cell lines were treated with 5-AzaCdR and TSA, respectively, or with 5-AzaCdR and TSA concomitantly to verify if the effect of the treatment with both drugs would be amplified. Treatment was started at 40% confluence. The compound 5-AzaCdR was added to the plate at 10 μM final concentration in 10 ml of RPMI medium enriched with 5% FBS and the cells were cultivated for 24 hours in a humidified incubator at 37°C with 5% CO_2_. Every 24 hours, the medium was changed with the addition of 5-AzaCdR at the same conditions. For the treatment with TSA, the compound was added to the plate at the final concentration of 40 nM and the cells were maintained at the same conditions described before. For the treatment with 5-AzaCdR and TSA, the cells were primarily treated with 5-AzaCdR and, after 96 hours the medium was removed and the TSA was added, as described before. Subsequently, total RNA was extracted from the cells following TRIzol^®^ recommended protocol.

### Quantitative Real Time PCR

Quantitative analysis of mRNA expression was performed using the Corbett Rotor-Gene 6000 system detection using Rotor-Gene kit Fast Syber Green PCR Master Mix^®^ (Qiagen, Dusseldorf, Germany).

### Pyrosequencing

Pyrosequencing assay was performed with PyroMark Gold Q24 Qiagen kit (catalog number 1055272), according to the manufacturer’s instructions.

### *Sirt1* stable silencing

The stable silencing of *Sirt1* was performed by MISSION^®^ shRNA Lentiviral Transduction Particles from Sigma-Aldrich (SHCLNV-NM_019812). Lentiviral particles were used to establish cell lines with knockdown of *Sirt1* or expressing the non-target shRNA sequence as a control. All transductions were performed using a MOI (multiplicity of infection) of 0.5, in the presence of 8 μg/mL polybrene. After transduction, puromycin-resistant cells were selected. The target silencing was checked by RT-qPCR.

## SUPPLEMENTARY MATERIALS FIGURES AND TABLES








