# Supplementary material for: SIRT1 regulates Mxd1 during malignant melanoma progression
Source: Oncotarget. 2017 Oct 3;8(70):114540–53. doi: 10.18632/oncotarget.21457 (PMC5777712; doi:10.18632/oncotarget.21457)
Supplement: Supplementary file 1 [file oncotarget-08-114540-s001.pdf]

SIRT1 regulates *Mxd1* during malignant melanoma progression

SUPPLEMENTARY MATERIALS

Gene ontology (GO) analysis

The GO analysis was performed to evaluate the mainly pathways that the closest genes to the peaks where SIRT1 has associated in normal and suspension condition were involved. For this, firstly we have used DAVID tool to obtain GO terms and *p*-values for each gene, according the procedure recommended (Huang et al., 2008). Then, we have used the obtained GO terms and *p*-values (less or equal to 0.05) for REVIGO analysis. Based on algorithms that reduce redundance, REVIGO finds a subset of representative and non-redundant GO terms as described previously (Supek et al., 2011). The results were represented by two scatterplots, one for adhesion and other for deadhesion condition, showing the representatives clusterings remaining after the redundance reduction. More semantically similar GO terms are closer in the plot. The bubble size indicates the frequency of the GO term, while the color represents the *p*-value according to legend.

REFERENCES

1. Huang DW, Sherman BT, Lempicki RA. Systematic and integrative analysis of large gene lists using DAVID Bioinformatics Resources. Nature Protoc. 2009; 4:44–57.
2. Huang DW, Sherman BT, Lempicki RA. Bioinformatics enrichment tools: paths toward the comprehensive functional analysis of large gene lists. Nucleic Acids Res. 2009; 37:1–13.
3. Supek F, Bošnjak M, Škunca N, Šmuc T. REVIGO summarizes and visualizes long lists of Gene Ontology terms. PLoS One. 2011. https://doi.org/10.1371/journal.pone.0021800

Supplementary Table 1: SIRT1 differentially associated peaks in adhered and deadhered melan-a melanocytes. From left to right, the table shows the chromosome where the peak is located, the start and end position of the DNA sequence, width, concentration in adhesion, concentration in deadhesion, fold change value, *p*-value, FDR value, gene symbol of the closest gene to the peak, the gene ID and the distance of the peak to the closest gene. To this analysis, we have considered as differentially associated the peaks whose FDR values were less or equal to 0.1 and fold change values above |1|. See Supplementary_Table_1

Supplementary Table 2: REVIGO's table view of the GO terms of genes related to SIRT1 in adherent melanocytes. From left to right, the table shows the term ID, the description of the term, the frequency that the term appears, the log10-*p*value, the uniqueness (that represents the similarity of a term in relation to the other term) and the dispensability (that compares a term to other semantically close terms), respectively. See Supplementary_Table_2

Supplementary Table 3: REVIGO's table view of the GO terms of genes related to SIRT1 under stress condition (deadhesion). From left to right, the table shows the term ID, the description of the term, the frequency that the term appears, the log10-*p*value, the uniqueness (that represents the similarity of a term in relation to the other term) and the dispensability (that compares a term to other semantically close terms), respectively. See Supplementary_Table_3
